# Collagen promotes PD-L1 overexpression in fibroblasts through binding to CD44 and activating YAP1 signaling during keloid formation

**DOI:** 10.1038/s41598-026-59337-6

**Published:** 2026-06-25

**Authors:** Lingfeng Chen, Yakun Gao, Sida Pan, Zhu Zhu, Jianlan Liu, Ningwen Zhu, Baojin Wu

**Affiliations:** 1 Department of Plastic Surgery, Huashan Hospital, Fudan University, Shanghai, 200040 China; 2 Department of Dermatology, Huashan Hospital, Fudan University, Shanghai, 200040 China; 3 Department of Plastic Surgery, Ninth People’s Hospital, Shanghai Jiao Tong University School of Medicine, Shanghai, 200011 China

**Keywords:** Keloid, Fibroblast, Collagen, CD44, PD-L1, YAP1 signaling, Cell biology, Diseases, Medical research, Molecular biology

## Abstract

Keloid is a skin collagen disease secondary to skin injury, characterized by excessive collagen deposition and a markedly high rate of recurrence. To date, there is still a lack of effective prevention and treatment methods for keloid. Thus, it is urgent to explore the main pathological mechanism of keloid formation, which requires to identify the biological mechanism of deposited collagen regulating the formation of fibroblast-mediated local immunosuppressive microenvironment. Collagen accumulation, CD4^+^ T, CD8^+^ T, CD86^+^ Macrophages, FOXP3^+^ Treg cells infiltration, CD44 expression, and α-smooth muscle actin (α-SMA), platelet-derived growth factor receptor (PDGFR) and programmed cell death 1 ligand 1 (PD-L1) levels in keloid and normal skin tissue samples were determined by tissue immunofluorescence assay. The effects of collagen I on the expression of PD-L1, YAP1 and phosphorylated YAP1 (p-YAP1) in primary keloid fibroblasts were examined by reverse transcription-quantitative PCR and western blot assays. The co-location of collagen I and CD44 or PD-L1 and α-SMA was determined by cell immunofluorescence assay. Collagen accumulated in keloid pathological tissues, and fibroblasts proliferated abnormally in keloid pathological tissues. The infiltration of CD4^+^ T, CD86^+^ Macrophages and FOXP3^+^ Treg cells in keloid tissues was significantly upregulated, while the infiltration of CD8^+^ T cells in keloid tissues was significantly reduced. PD-L1 was significantly overexpressed in the fibroblasts of keloid tissues. Further mechanistic exploration found that deposited collagen (induced by inflammation) could bind to CD44 on the surface of fibroblasts, activate the YAP1 signaling pathway and lead to PD-L1 overexpression to form a local immunosuppressive microenvironment, resulting in the inability to remove the deposited collagen and the continuous proliferation of fibroblasts, ultimately forming keloid. The present study revealed that collagen deposition induced an immunosuppressive microenvironment and promoted the formation of keloid, thus providing potential new targets and theoretical basis for the prevention and treatment of keloid.

**Supplementary Information:**

The online version contains supplementary material available at 10.1038/s41598-026-59337-6.

## Introduction

Keloid is a skin collagen disease secondary to skin injury such as trauma, infection, burn or surgery, which is characterized by excessive collagen deposition in the dermis and subcutaneous tissue^1,2^. Currently, whether plastic surgery or dermatology, the existing treatment methods for keloid, have a high recurrence rate. The recurrence rate of simple resection is as high as 55–100%, even inducing more serious keloid, which causes considerable pain in patients^3,4^. The pathogenesis of keloid is not clear at present. With the deepening of research, keloid is considered to be caused by multiple factors, including activation of oncogenes, functional inactivation of tumor suppressor genes, genetic and epigenetic changes of tumor-related genes, and external stimuli^5^. Moreover, keloid is not only a local disease, but its pathogenesis also involves systemic immunological abnormalities^6–11^. Therefore, it is of great scientific importance and clinical value to deeply study the biological mechanism behind the formation of keloid in order to improve the clinical prevention and treatment of keloid, and to develop effective targeted drugs for keloid.

The common pathological characteristics of keloid are abnormal proliferation of a large number of fibroblasts, excessive deposition of extracellular matrix protein and disordered arrangement of collagen fibers^12^. During keloid formation, keloid fibroblasts show strong proliferative potential, and are more sensitive to transforming growth factor-β1 (TGF-β1) and platelet-derived growth factor (PDGF), causing high expression of collagen (including collagen type I) and extracellular matrix (ECM)-associated genes, and increased accumulation of collagen and ECM^13,14^. Our preliminary study showed that abnormal proliferation of fibroblasts was the fundamental reason for the continuous progression and recurrence of keloid. Of note, α-smooth muscle actin (α-SMA) and platelet-derived growth factor receptor (PDGFR) are the two most common indicators for identifying fibroblasts. From the perspective of pathobiology, keloid is more similar to a chronic inflammatory disease with tumor tendency. Regardless of the type of inflammation induced by the initial stimulation, from the perspective of the cellular immune microenvironment generated by keloid, the regulation of local inflammatory response on fibroblast proliferation and collagen secretion plays a key role in the process of scar formation^1^. In a previous study, collagen deposition could lead to a decrease in the total number of T cells and T cell failure, leading to resistance to programmed cell death 1 ligand 1 (PD-L1) blockers^15^. Our preliminary study suggested that there was a large quantity of collagen deposition in keloid tissue, and the total number of infiltrated CD8^+^ T cells was reduced, suggesting that the increase in collagen deposition may be closely related to the imbalance of local immune microenvironment.

It has been reported that collagen type I could bind to CD44^16,17^, which is commonly detected on the surface of lymphocytes and fibroblasts. The CD44-mediated activation of signaling pathways has shown a strong association with the abnormal proliferation of fibroblasts^16–19^. Moreover, CD44 could activate the YAP signaling pathway, thereby activating the expression of downstream target genes such as PD-L1^20,21^. However, it is unknown whether this mechanism plays a key role in the formation of keloid.

Therefore, this present study mainly investigated the biological mechanism of deposited collagen regulating the formation of fibroblast-mediated local immunosuppressive microenvironment, which caused the inability of clearing deposited collagen and the continuous proliferation of fibroblasts, which finally formed a keloid. The current findings provide a scientific basis for the development of more effective prevention methods and drugs for the treatment of keloid.

## Materials and methods

### Collection of clinical samples

**Fig. 1:**
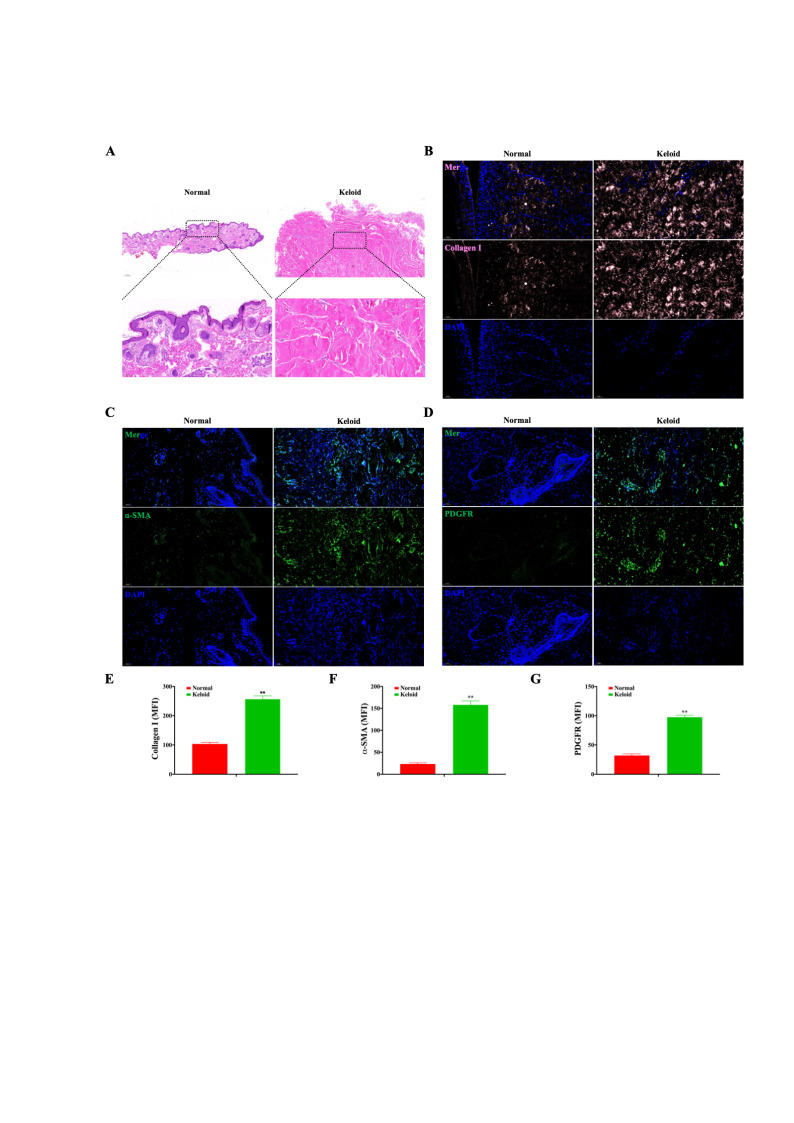
Collagen deposition and abnormal proliferation of fibroblasts are the main causes of the continuous progression and recurrence of keloid. (**A**) HE staining of the keloid pathological tissues and the normal skin tissues. (**B, E**) Immunofluorescence detection and quantitative assay of collagen I in keloid pathological tissues and normal skin tissues. (**C, F**) Immunofluorescence detection and quantitative assay of α-SMA in keloid pathological tissues and normal skin tissues. (**D, G**) Immunofluorescence detection and quantitative assay of PDGFR in keloid pathological tissues and normal skin tissues. Here, all keloid tissues (*n* = 60) and normal scar tissues (*n* = 30)were stained with antibodies against Collagen I, α-SMA, PDGFR, respectively. Nuclear staining was done with DAPI. Scale bar, 50 μm. Student’s t-test was used to analyze the data. *P < 0.05; **P < 0.01.

This clinical study was approved by the Ethics Committee of Huashan Hospital Affiliated to Fudan University (Shanghai, China; approval no. 2022 Clinical Review No. 913), and each patient signed an informed consent before enrolling in this study. Research involving human research participants have been performed in accordance with the Declaration of Helsinki. All clinical tissues were obtained from patients undergoing surgical treatment in Huashan Hospital Affiliated to Fudan University during 1 January 2023 and 31 December 2024. The patients provided written informed consent for publication of the representative keloid images in Fig. 1A. In the recruited 60 patients, the number of males and females were 28 and 32, respectively. The age range was between 14 and 72, with the median age 31. For minors (patients < 18 years old), their parents/guardians provided consent for participation. Informed consent were provided from all subjects and/or their legal guardian(s), for both study participation and publication of identifying information/images in an online open-access publication. All keloid tissues (*n* = 60) were collected from patients confirmed to have clinical evidence of keloid. No keloid patient received chemotherapy, radiotherapy, or intralesional steroids treatment prior to surgery. All keloid tissues were obtained by removing the scar through excision, and the scar used is preferably mature scar for more than one year, while normal skin tissue is taken from patients who undergo body contouring surgery to remove excess skin. All normal scar tissues (*n* = 30) were obtained from patients who underwent elective scar resection surgery.

### HE staining and immunohistochemistry

Paraffin-embedded tissues were sectioned for HE (hematoxylin and eosin) and IHC (immunohistochemistry) staining. For IHC analysis, the experiments were performed using the first antibody, HRP-conjugated secondary antibody, and DAB (diaminobenzidine) detection reagents. The DMI3000B microscope connected to the digital imaging system was applied for taking photographs and the following analysis. All the data were evaluated and classified blindly by two investigators.

### Tissue immunofluorescence

Immunofluorescence staining was performed on formalin-fixed paraffin-embedded human keloid and normal scar biopsies. Tissue sections were deparaffinized and rehydrated followed by heat-induced antigen retrieval in citrate buffer at pH 6.0 for 10 min. Antibodies were applied, including mouse anti-α-SMA (BF9212, Affinity Biosciences) (1:100), rabbit anti-Collagen I (ab138492, Abcam), rabbit anti-CD4 (ab133616, Abcam), rabbit anti-CD8 (ab316778, Abcam) (1:100), rabbit anti-CD68 (ab283654, Abcam), mouse anti-FOXP3 (ab36607, Abcam), or mouse anti-PD-L1 (ab279292, Abcam) (1:100) were incubated overnight at 4 °C. Sections were washed with PBS three times, and then labeled with Alexa Fluor 488 (A11029, ThermoFisher) and 647 (A31634, ThermoFisher) labeled secondary antibodies (1:5000). Slides were cover slipped using DAPI containing aqueous mounting medium. All the tissues immunofluorescence high-resolution panoramic images were obtained through the OLYMPUS VS200 digital pathology slide scanner. ImageJ software was applied for quantitative analysis of target protein expression.

### Isolation and identification of primary fibroblasts

Human skin biopsies were obtained from human keloid. The skin tissues were plated in the culture dish with Dulbecco’s modified Eagle’s medium (DMEM; Gibco BRL, Grand Island, NY, USA), and primary fibroblasts were established as described previously^22^. Primary fibroblasts were grown in DMEM supplemented with 10% fetal bovine serum (PAA Laboratories, Etobicoke, Ontario, Canada), 100 U/mL penicillin, and 100 µg/mL streptomycin (Gibco BRL) at 37 ^◦^C in a humidified atmosphere containing 5% CO_2_. When confluent, the primary fibroblasts were trypsinized using a 0.25% trypsin/0.02% EDTA solution (Sigma, St Louis, MO, USA) and subcultured at a 1:3 ratio on culture plastic dishes. After three or six passages, the primary fibroblasts were used for the following experiments. In this study, we extracted fresh fibroblasts for each experiment and did not perform immortalization.

### In vitro collagen intervention on primary fibroblasts

24-well cell culture plates (Corning, USA) were pre-treated by coating with human type I collagen (Cat. No. C2249, Sigma-Aldrich, USA) dissolved in 0.1 mol/L acetic acid. Three coating concentrations were prepared to simulate varying collagen deposition levels: high (15 µg/cm²), medium (10 µg/cm²), and low (5 µg/cm²). Plates were incubated overnight at 4 °C to allow sufficient collagen adsorption onto the plastic surface. The following day, residual acetic acid was thoroughly removed by washing each plate three times with pre-cooled Hanks’ Balanced Salt Solution (HBSS). Non-specific binding sites on the plate surface were then blocked with 0.1% (w/v) bovine serum albumin (BSA) dissolved in high-glucose Dulbecco’s Modified Eagle Medium (DMEM) for 1 h at room temperature. After blocking, plates were washed another three times with HBSS to eliminate residual BSA solution. Control group plates underwent identical BSA blocking treatment without prior collagen coating, as described in a previous study^23^.

Human primary fibroblasts were isolated from surgically excised keloid tissues using established enzymatic digestion and differential adhesion methods. For the co-culture experiment, fibroblasts were seeded at a density of 5 × 10⁴ cells per well onto type I collagen-coated plates (high: 15 µg/cm², medium: 10 µg/cm², low: 5 µg/cm²) and uncoated control plates. This experimental design aimed to mimic the heterogeneous collagen microenvironment characteristic of keloid tissues. All cultures were maintained in a humidified incubator at 37 °C with 5% CO₂ for 48 h to allow cell-matrix interactions. After the incubation period, adherent fibroblasts were harvested using 0.25% trypsin-EDTA solution and centrifuged at 1,200 rpm for 5 min. Cell pellets were then resuspended in fresh DMEM for subsequent functional assays.

### Plasmids construction and transfection

Three shRNA fragments for human CD44 were synthesized by the Sangon Biotech company (Shanghai, China), sub-cloned into the pLKD shRNA vector, and named pLKD-CMV-G&PR-U6-CD44-shRNA1/2/3, respectively. The shRNA sequences were listed as follow: shRNA1: 5’-TTCAAATCGATCTGCGCCAGGC-3’; shRNA2: 5’-TATATTCAAATCGATCTGCGCC-3’; shRNA3: 5’-TATTGAAAGCCTTGCAGAGGTC-3’; shRNA-NC: 5’- TTCTCCGAACGTGTCACGTTT-3’. All the plasmids were used to transfect the primary fibroblasts by using Lipofectamine 3000 transfection reagent.

### Real time PCR

Real-time quantitative PCR was performed as previously described^24^. Briefly, Total RNA was isolated with TRIzol reagent (Invitrogen). First-strand cDNA was synthesized using a PrimeScript RT Reagent Kit (Takara, Japan). Gene-specific primer pairs were designed with Primer Premier 5.0 software, and the primer sequences were listed as follow: PD-L1, forward primer: 5-TTTGCTGAACGCCCCATACA-3, reverse primer: 5-TGTCCCGTTCCAACACTGAG-3; CD44, forward primer: 5-ACTAAATCAGGGCTGGGCTT-3, reverse primer: 5-GAGAGGGTAGACAGGGAGGA-3; GAPDH, forward primer: 5- GGTGGTCTCCTCTGACTTCAACA-3, reverse primer: 5-CCAAATTCGTTGTCATACCAGGAAATG-3. Quantitative real-time PCR was performed using SYBR Green Master (Takara, Japan) according to the manufacturer’s protocol.

### Western blot

Western blot was performed as previously described^22^. Briefly, cells were lysed in 1×SDS-PAGE loading buffer (50 mM Tris-HCl at pH 6.8, 2% (W/V) SDS, 0.1% (W/V) BPB, 10% (V/V) glycerol) containing 50 mM β-glycerophosphate, and the lysates were resolved by SDS-PAGE and transferred to polyvinylidene difluoride membranes for western blot using enhanced chemiluminescence detection reagents (Merck Millipore, Billerica, MA). The primary antibodies were beta-actin (1:1,000, AF0003, Beyotime), α-SMA (1:1,000, BF9212, Affinity Biosciences) and PD-L1 (1:1,000, ab279292, Abcam), YAP1 (1:1,000, 14074, CST), p-YAP1 (1:1,000, 13008, CST), and the secondary antibody was horseradish peroxidase- conjugated goat anti-mouse (1:10,000, Abcam) or goat anti-rabbit. Protein expression was observed and visualized by chemiluminescence using an Alpha Imager scanner (Tecan, Thermo Scientific). The ImageJ software was utilized for the quantification of protein expression in Western blots.

### Cellular immunofluorescence

For cell samples, the culture medium was aspirated, and the cells were washed twice with pre-cooled PBS at 4 °C, and fixed by 4% paraformaldehyde overnight at 4 °C. For immunofluorescence staining, cells were permeabilized with 0.25% Triton X-100, blocked with 2% BSA, and incubated with PD-L1 (1:100, ab279292, Abcam) or α-SMA (1:100, BF9212, Affinity Biosciences) at 4 °C overnight, washed, and incubated with appropriate amount of Alexa Fluor 488 or Alexa Fluor 647-conjugated secondary antibody (1:1000, Abcam, ab150079, USA) at ambient temperature for 1.5 h, Then, the cells were counterstained with DAPI (Thermo Fisher, 62248, USA) for 10 min, mounted with anti-quenching mounting medium, and stained. The results were observed and stored using a DMI3000B microscope (Leica, Germany).

### Flow cytometry assay of the fibroblast on T cell proliferation and activation

For the isolation of primary fibroblasts, a total of 20 male C57BL/6 mice (4-6 weeks old; 18–20 g) were purchased from SLAC Laboratory Lab (Shanghai, China). All the mice were given free access to food and water and kept in an SPF environment with the temperature between 24 ± 2 °C, the relative humidity was 60%, and 12‐h light/dark cycle. All the experimental procedures were approved by the Ethics Committee of Huashan Hospital Affiliated to Fudan University (Shanghai, China; approval no. 2023-HSYY-004JZS) and adhered to the national institutes of Health Guide for the care and use of animal guidelines. All experiments were performed in accordance with the American Veterinary Medical Association (AVMA) Guidelines for the Euthanasia of Animals (2020), as a comprehensive resource for guidance on veterinary best practice for the anaesthesia and euthanasia of animals. All the authors comply with the IUCN Policy Statement on Research Involving Species at Risk of Extinction and the Convention on the Trade in Endangered Species of Wild Fauna and Flora. The mice were anesthetized with 40 mg/kg pentobarbital sodium solution by intraperitoneal injection. Then, the mice were sacrificed by cervical dislocation and the skin tissues were subsequently collected. No mice were sacrificed due to humane endpoints and no mice were found lifeless throughout the experiment. The skin tissues were cut into 3 mm strips and incubated in Dispase (0.05%) and DMEM at 4 ◦C overnight. Next, the epidermis was removed from the dermis. The dermis was minced finely and placed appropriately in plastic dishes (in advance-coated with FBS), and placed horizontally for one hour and then vertically for three hours in an atmosphere of 5% CO_2_ at 37 °C. The tissues were cultured in DMEM supplemented with 5.5 mM glucose, 10% FBS and 1% penicillin-streptomycin, following by medium change every 3 days. The cultured cells were digested and passaged with 0.25% trypsin after the cell confluence reached approximately 80%. After three passages, the primary fibroblasts were used for the following experiments.

For the isolation of primary T cells, as above, collect mouse spleen tissues, take one sterile 1 mL syringe, remove the syringe cap, clamp the spleen with tweezers, draw 0.5% BSA-PBS solution with the 1 mL syringe, repeatedly blow the spleen, and collect the blown cell suspension. 200 g, 4 ◦C, centrifuge for 10 min, discard the supernatant, add 3 mL of sterile red blood cell lysis buffer pre-cooled at 4 ◦C, gently blow with a pipette, lyse the cells at 4 ◦C for 3 min until the solution becomes clear and transparent, add 6 mL of sterile PBS to terminate cell lysis, and filter the cell suspension into a new centrifuge tube using a 70 µM cell sieve. 200 g, 4 ◦C, centrifuge for 10 min, discard the supernatant, and resuspend the cells in 5 mL sterile PBS to form a single-cell suspension. 200 g, 4 ◦C, centrifuge for 10 min, discard the supernatant, add 20 µL buffer to every 10^7^ cells, and mix the cells well. Add 5 µL Biotin Antibody Cocktail to every 10^7^ cells, mix the cells well, and incubate at 4 ◦C for 5 min. Add 15 µL buffer for every 10^7^ cells. Add 10 µL of Anti Biotin MicroBeans to every 10^7^ cells, mix the cells well, and incubate at 4 ◦C for 10 min. Finally, use a sorting magnetic frame to clamp the LS column, add the mixed cells into the LS column, collect the obtained T cell suspension, and leave it for subsequent co-culture experiments.

The isolated primary fibroblasts were co-cultured with primary T cells of the same genus at a ratio of 1:10 in 96 well cell culture plates until the 4th generation in vitro. Since primary T cells are suspension-cultured, we directly collected the co-culture supernatant, centrifuged to obtain the T cells, and resuspended them in PBS for subsequent experiments. On the one hand, T cells were stained with CFSE for T cell proliferation assay at three co-culture time points of 48 h, 72 h, and 96 h. Specifically, CFSE-labeled T cells were assessed by flow cytometry based on the sequential halving of CFSE fluorescence intensity with each cell division. The multiple peaks correspond to successive generations of divided T cells, with each peak representing one round of division. Proliferation was assessed by analyzing all peaks corresponding to divided cells (i.e., peaks with reduced CFSE fluorescence compared to the undivided parent population). The percentage of proliferated cells was calculated by gating on all divided peaks, excluding the undivided peak (peak 0). On the other hand, as before, primary fibroblasts and T cells were co-cultured in a 96 well cell culture plate for 120 h. Then, T cells were labeled and stained with CD69 antibody, and the activation of T cells in each group was detected by flow cytometry.

### Statistical analysis

All data were expressed as the mean ± standard deviation. For normally distributed data, significance of mean differences was determined by unpaired Student’s t-tests. For groups that differed in variance, unpaired t-test with Welch’s correction was performed. One-way analysis of variance was used to analyze differences among three or more groups, followed by Tukey’s post hoc analyses for between‐group comparisons. All statistical analyses were performed using GraphPad Prism 9 software and SPSS (version 26.0). A two-tailed *P* value of 0.05 or less was considered statistically significant.

## Results

### Collagen deposition and abnormal proliferation of fibroblasts are the main causes of the continuous progression and recurrence of keloid

Keloid pathological tissues were collected from patients with keloid in the earlobe or perineum. Hematoxylin and eosin staining showed that, compared with normal skin tissues, the number of fibroblast-like cells was significantly increased in the pathological tissues of keloid (Fig. 1A). Subsequently, the expression of collagen in keloid pathological tissues, and found that compared with normal skin tissue, the expression of collagen I in keloid pathological tissue increased significantly (Fig. 1B and E), suggesting that the deposition of collagen is related to the formation of keloid. Next, to further verify the presence of fibroblasts in keloid tissues, the expression level of fibroblast-specific proteins such as α-SMA and PDGFR was detected in keloid tissues by immunofluorescence methods. The results showed that, compared with normal skin tissues, both α-SMA and PDGFR exhibited a higher level of expression in keloid pathological tissues (Fig. 1C, D, F and G). These results suggest that deposition of collagen and abnormal proliferation of fibroblasts in keloid tissues are closely related to the continuous progression of keloid.

### Abnormal proliferation of fibroblasts is accompanied by local immunosuppressive microenvironment phenomena

**Fig. 2:**
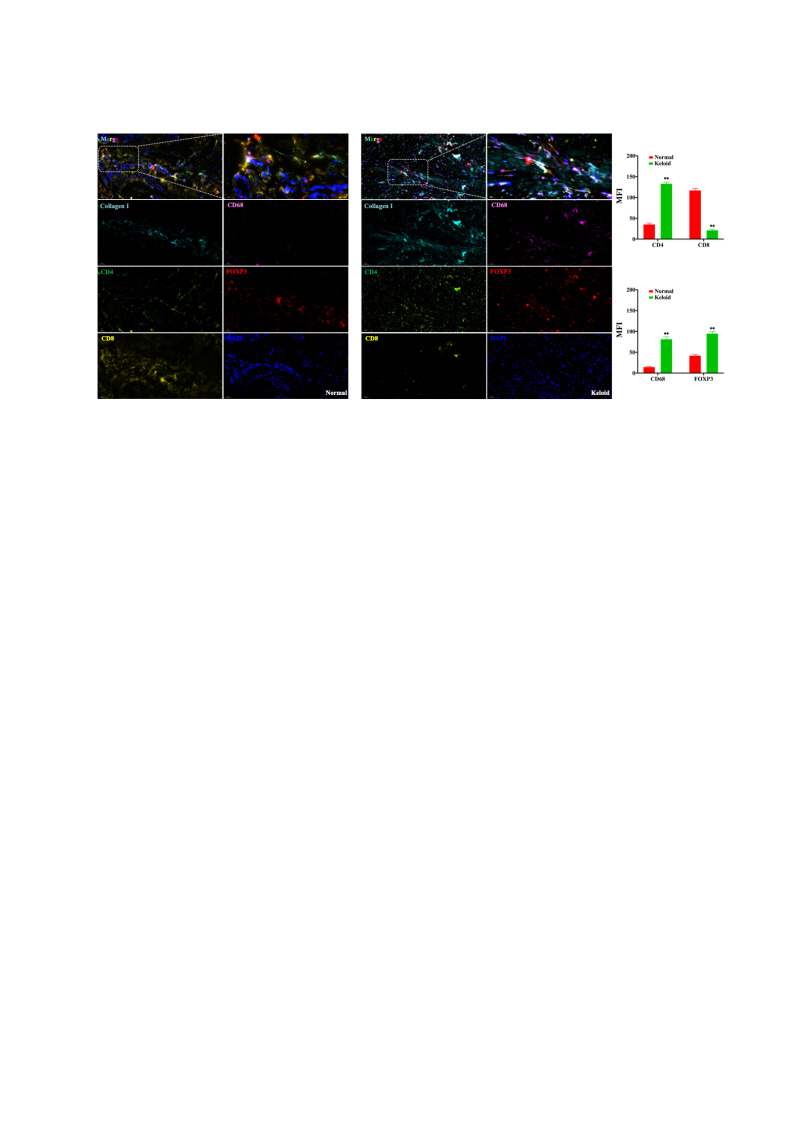
Abnormal proliferation of fibroblasts is accompanied by local immunosuppressive microenvironment phenomena. Multi-immunofluorescence detection and quantitative assay of collagen I, CD4, CD8, CD68 and FOXP3 in keloid pathological tissues (*n* = 12) and normal skin tissues (*n* = 6). The tissues were stained with antibodies against collagen I, CD4, CD8, CD68 and FOXP3, respectively. Nuclear staining was done with DAPI. Scale bar, 50 μm and 20 μm Student’s t-test was used to analyze the data. *P < 0.05; **P < 0.01.

To explore whether there is an association between fibrous dysplasia and immunosuppressive microenvironment, the multiple immunofluorescence experiments were applied to examine the immune landscape in keloids tissues, including the immune markers CD4, CD8, CD68 and FOXP3, as well as Collagen I. Our multiplex immunofluorescence results show that the expression of CD4, CD68, and FOXP3 in keloid tissue is significantly higher than in normal skin tissue, whereas CD8 expression is significantly lower in keloid tissue (Fig. 2).‌ This suggests that CD4^+^ T cells, macrophages, and Treg cells are markedly infiltrated in keloid tissue, while CD8^+^ T cell infiltration is notably reduced, contributing to an immunosuppressive microenvironment in keloids. Notably, although Treg cells typically suppress inflammation, their function may be “reprogrammed” within the keloid microenvironment to promote fibrosis and abnormal collagen accumulation.

### High expression of PD-L1 in fibroblasts of keloid tissues

**Fig. 3:**
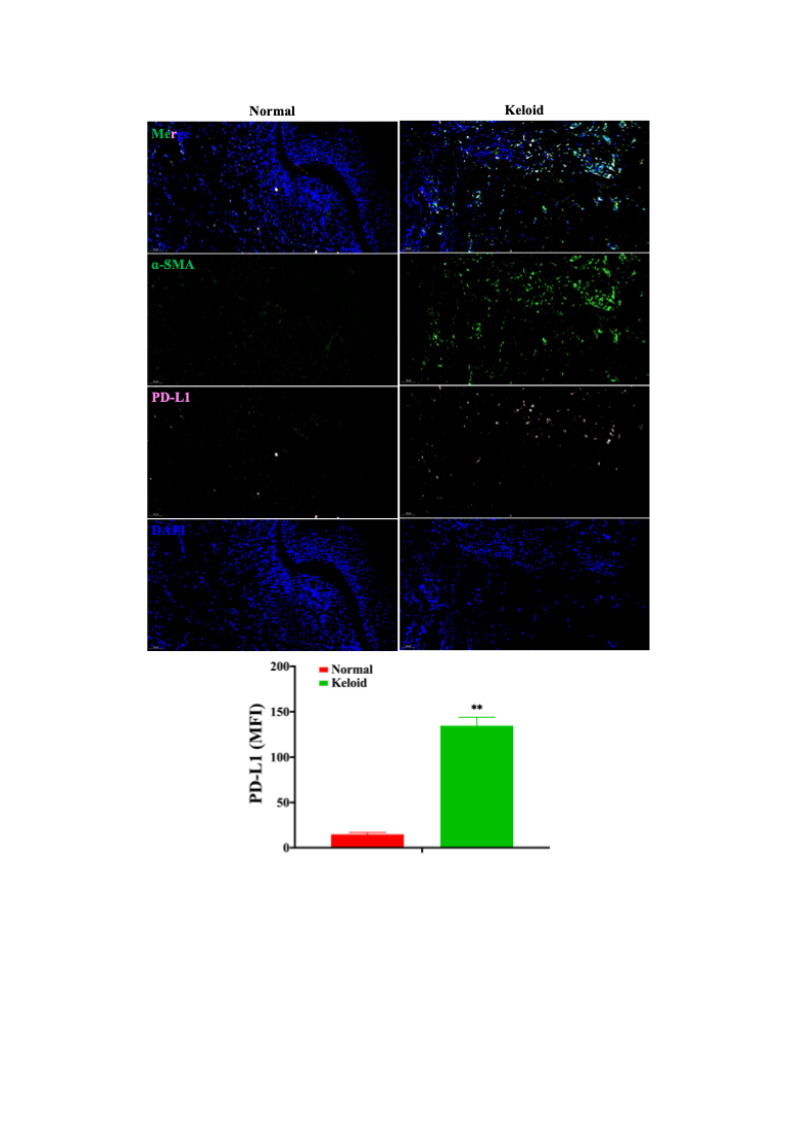
High expression of PD-L1 in fibroblasts of keloid tissues. Immunofluorescence detection and quantitative assay of α-SMA and PD-L1 in keloid pathological tissues (*n* = 12) and normal skin tissues (*n* = 6). The tissues were stained with antibodies against α-SMA and PD-L1, respectively. Nuclear staining was done with DAPI. Scale bar, 50 μm. Student’s t-test was used to analyze the data. *P < 0.05; **P < 0.01.

As known, PD-L1 is an inhibitory ligand that normally plays a role in maintaining immune homeostasis. When diseases occur, certain cells overexpress PD-L1 molecules, which bind to receptors B7.1 and PD-1 on the surface of activated T cells, causing T cells to lose their cytotoxic activity and interfering with the body’s immune response^25^. Therefore, the expression of PD-L1 in keloid tissues was then determined in the present study. The immunofluorescence results showed that PD-L1 and α-SMA exhibited notable co-localization, and compared with normal skin tissue, PD-L1 was significantly overexpressed in fibroblasts of keloid pathological tissue (Fig. 3). These results suggest that overexpression of PD-L1 by fibroblasts with abnormal hyperplasia in keloid tissue is an important part of the formation of an immunosuppressive microenvironment.

### Co-expression of collagen and collagen receptor CD44 on fibroblast surface in keloid

**Fig. 4:**
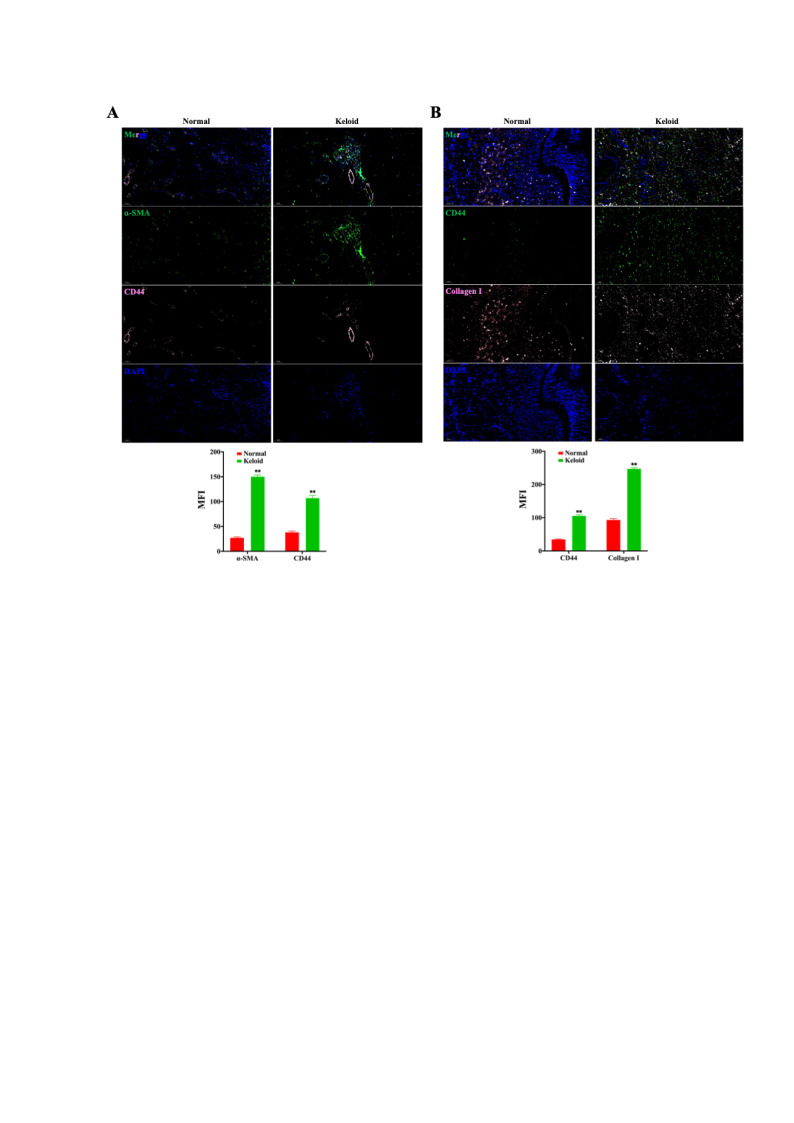
Co-expression of collagen and collagen receptor CD44 on fibroblast surface in keloid. (**A**) Immunofluorescence detection and quantitative assay of α-SMA and CD44 in keloid pathological tissues (*n* = 12) and normal skin tissues (*n* = 6). The tissues were stained with antibodies against α-SMA and CD44, respectively. Nuclear staining was done with DAPI. Scale bar, 50 μm. (**B**) Immunofluorescence detection and quantitative assay of CD44 and collagen I in keloid pathological tissues (*n* = 12) and normal skin tissues (*n* = 6). The tissues were stained with antibodies against CD44 and collagen I, respectively. Nuclear staining was done with DAPI. Scale bar, 50 μm. Student’s t-test was used to analyze the data. *P < 0.05; **P < 0.01.

Previous studies have suggested that PD-L1 is overexpressed by fibroblasts with abnormal hyperplasia in keloid tissue. Previous studies have shown that collagen receptor CD44 can promote the overexpression of the target gene PD-L1^26^. Therefore, the expression of CD44 on the membrane of fibroblasts in keloid tissue was further investigated in the present study. The immunofluorescence results showed that CD44 exhibited a high level of expression on the fibroblast membrane of normal skin and keloid tissues (Fig. 4A). Furthermore, it was found that collagen I and CD44 were highly co-localized in keloid tissue compared with normal skin tissue (Fig. 4B). The results preliminarily suggest that collagen in keloid tissue can directly interact with CD44 on the surface of fibroblasts to affect the expression of downstream target genes.

### Collagen I binds to CD44 on the surface of fibroblasts and promotes PD-L1 overexpression in fibroblasts

**Fig. 5:**
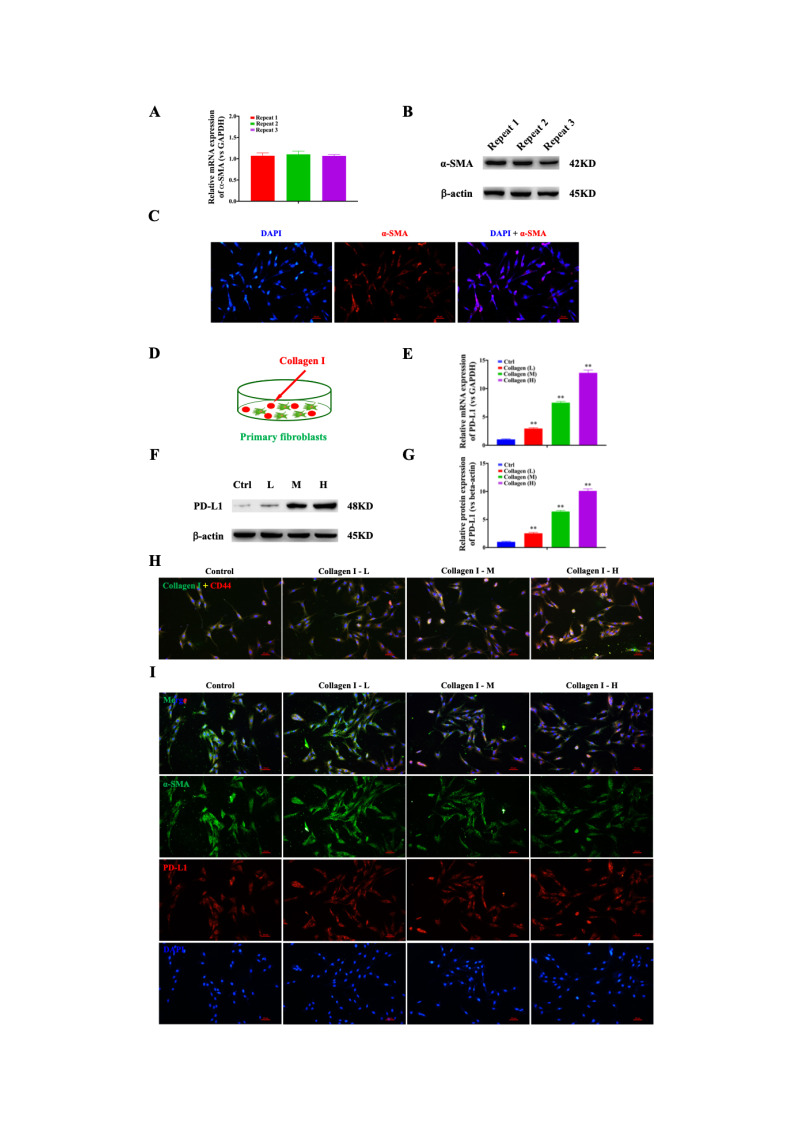
Collagen I binds to CD44 on the surface of fibroblasts and promotes PD-L1 overexpression in fibroblasts. In this section, the primary fibroblasts were isolated from the human skin biopsies obtained from human keloid. (**A**) Real time PCR assay of α-SMA mRNA in primary fibroblasts. Repeat 1, Repeat 2 and Repeat 3 represent three repeated experiments. (**B**) Western blot assay of α-SMA protein in primary fibroblasts. Repeat 1, Repeat 2 and Repeat 3 represent three repeated experiments. (**C**) Immunofluorescence detection of α-SMA in primary fibroblasts. (**D**) Co-incubation of collagen I and primary fibroblasts. (**E**) Real time PCR assay of PD-L1 mRNA in primary fibroblasts that co-cultured with collagen I of different concentration (Low, Middle, High). (**F,G**) Western blot and quantitative assay of PD-L1 protein in primary fibroblasts that co-cultured with collagen I of different concentration (Low, Middle, High). (**H**) Immunofluorescence detection of collagen I and CD44 in primary fibroblasts that co-cultured with collagen I of different concentration (Low, Middle, High). The cells were stained with antibodies against collagen I and CD44, respectively. Nuclear staining was done with DAPI. Scale bar, 50 μm. (**I**) Immunofluorescence detection of PD-L1 and α-SMA in primary fibroblasts that co-cultured with collagen I of different concentration (Low, Middle, High). The cells were stained with antibodies against PD-L1 and α-SMA, respectively. Nuclear staining was done with DAPI. Scale bar, 50 μm. Each experiment was performed three times independently. All results were shown as mean ± SD. Student’s t-test was used to analyze the data. **, *P* < 0.01.

Literature mining suggests that, collagen could bind to CD44 to activate the YAP1 signaling pathway and promote the expression of downstream genes, such as PD-L1 (Figure S1). Therefore, based on the aforementioned results, primary fibroblasts were further utilized in the present study to investigate the potential molecular mechanisms during the formation of keloid. First, primary fibroblasts were isolated from skin tissue, and PCR, western blot and immunofluorescence identification were performed (Fig. 5A-C). Next, fibroblasts were seeded at a density of 5 × 10⁴ cells per well onto type I collagen-coated plates (high: 15 µg/cm², medium: 10 µg/cm², low: 5 µg/cm²) and uncoated control plates. (Fig. 5D). Subsequent PCR and western blot results showed that collagen I significantly promoted the mRNA and protein expression of PD-L1 in fibroblasts in a dose-dependent manner (Fig. 5E, F and G). Furthermore, co-localization of collagen I and CD44, as well as co-expression of PD-L1 and α-SMA, were observed by cellular immunofluorescence. As expected, collagen I showed a high degree of co-localization with CD44 (Fig. 5H), and collagen I significantly promoted the protein expression of PD-L1 in fibroblasts (Fig. 5I). These results suggest that collagen can bind CD44 on the surface of fibroblasts and promote PD-L1 overexpression in fibroblasts.

### Collagen I binds to fibroblasts to induce an immunosuppressive microenvironment

**Fig. 6:**
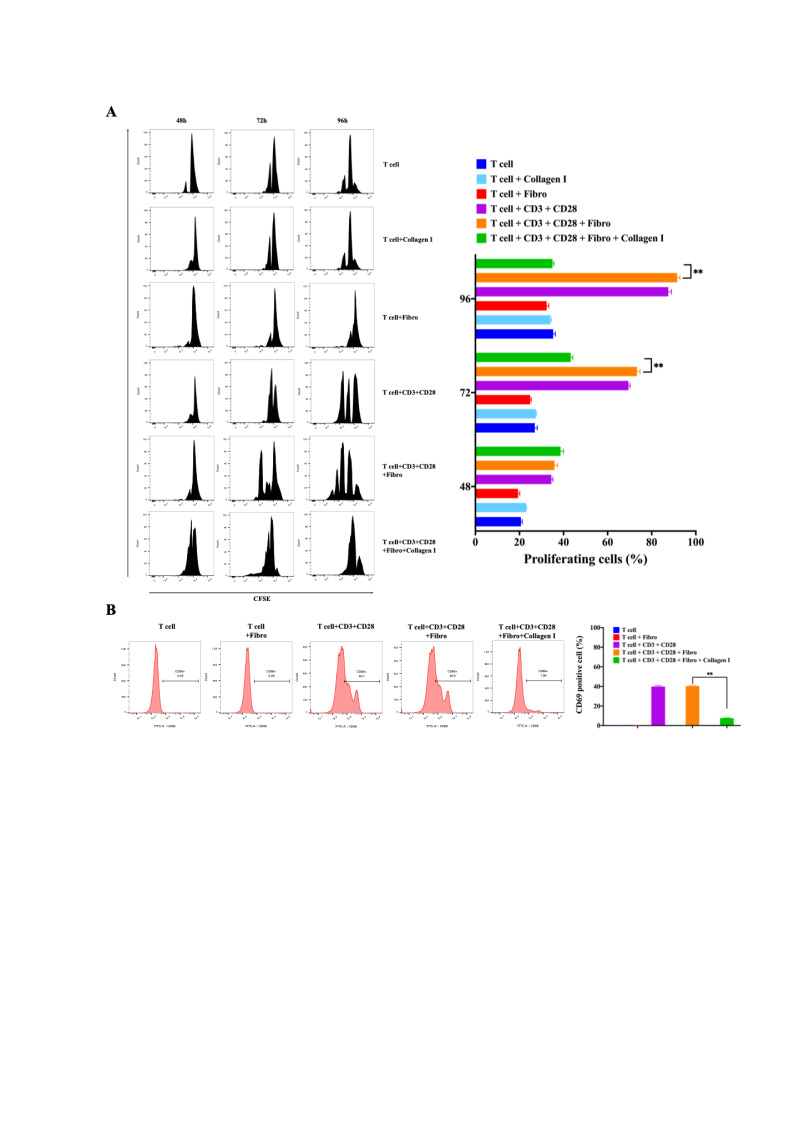
Collagen I binds to fibroblasts to induce an immunosuppressive microenvironment. In thise section, the primary fibroblasts were isolated from the skin tissues of C57BL/6 mice, and the primary T cells were isolated from the spleen tissues of C57BL/6 mice. (**A**) Fibroblasts inhibit the proliferation of T cells. In a 96 well cell culture plate, T cells were co cultured with fibroblasts for 48 h, 72 h, and 96 h. CD3 and CD28 were used to stimulate T cells proliferation. The co-cultured cells were divided into six groups as indicated in the images. Flow cytometry was used to analyze the proliferation of T cells by staining T cells with CFSE. (**B**) Fibroblasts inhibit the activation of T cells. In a 96-well cell culture plate, T cells were co cultured with fibroblasts for 120 h. The co-cultured cells were divided into five groups as indicated in the images. Flow cytometry was used to analyze the activation of T cells by detecting the CD69 expression on the surface of T cells. Each experiment was performed three times independently. All results were shown as mean ± SD. Student’s t-test was used to analyze the data. **, *P* < 0.01.

Subsequently, the present study further utilized primary fibroblasts and T cells to investigate whether collagen I affected T cell proliferation and activation via binding to fibroblasts. In a 96-well cell culture plate, simultaneous administration of the first and second signals CD3 and CD28 to T cells activated the initial T cells. Compared with the co-culture group of fibroblasts and T cells without collagen I, the proliferation ratio of T cells in the co-culture group of fibroblasts and T cells containing collagen I was significantly reduced, and the differences were statistically significant at 72 and 96 h, indicating that collagen I inhibits T cell proliferation by binding to fibroblasts (Fig. 6A). It should be specially noted that collagen I alone does not significantly affect T-cell proliferation, and the inhibitory effect requires the participation of fibroblasts (Fig. 6A). Similarly, compared with the co-culture group of fibroblasts and T cells without collagen I, the activation ratio of T cells in the co-culture group of fibroblasts and T cells containing collagen I was significantly reduced, and the differences were statistically significant, indicating that collagen I inhibits T cell activation by binding to fibroblasts (Fig. 6B). Overall, the above data indicate that collagen I can induce the formation of an immunosuppressive microenvironment by binding to fibroblasts.

### Collagen I promotes PD-L1 overexpression through activating YAP1 signaling

**Fig. 7:**
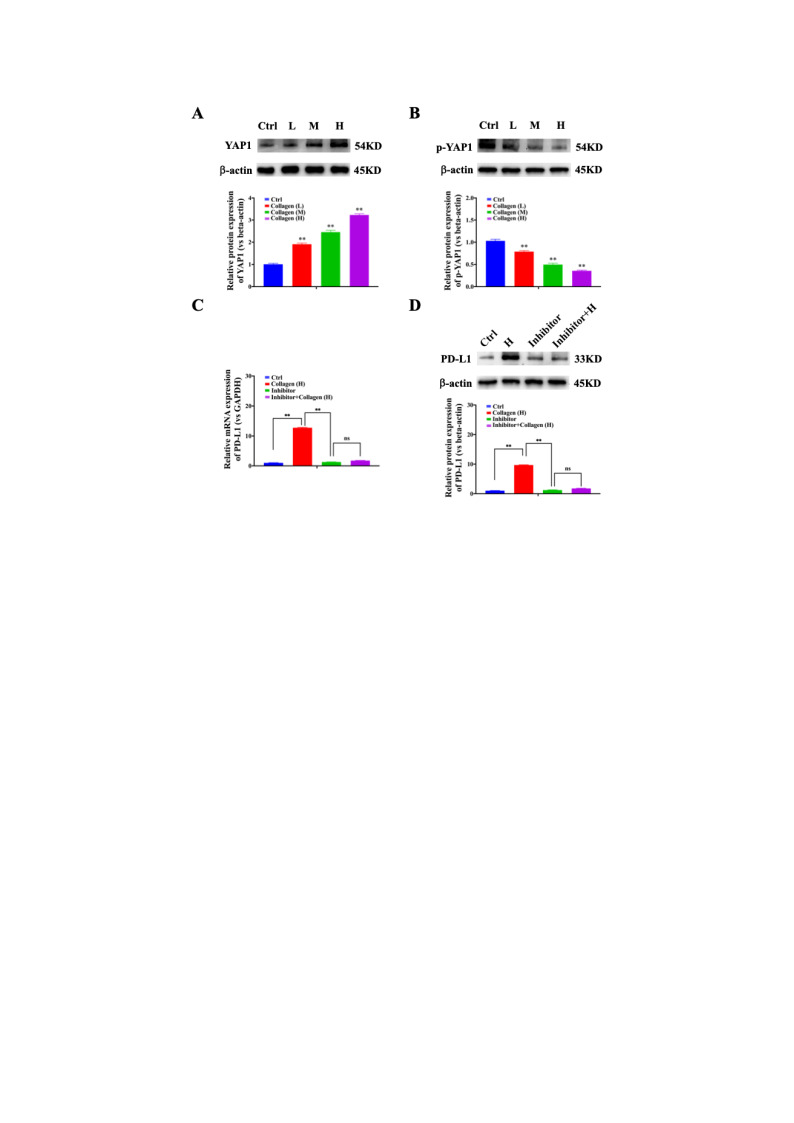
Collagen I promotes PD-L1 overexpression through activating YAP1 signaling. In this section, the primary fibroblasts were isolated from the human skin biopsies obtained from human keloid. (**A**) Western blot and quantitative assay of YAP1 protein in primary fibroblasts that co-cultured with collagen I of different concentration (Low, Middle, High). (**B**) Western blot and quantitative assay of p-YAP1 protein in primary fibroblasts that co-cultured with collagen I of different concentration (Low, Middle, High). (**C**) Real time PCR assay of PD-L1 mRNA in primary fibroblasts that co-cultured with high concentration collagen I with or without YAP1 signaling inhibitor (Cytochalasin D). (**D**) Western blot and quantitative assay of PD-L1 protein in primary fibroblasts that co-cultured with high concentration collagen I with or without YAP1 signaling inhibitor (Cytochalasin D). Each experiment was performed three times independently. All results were shown as mean ± SD. Student’s t-test was used to analyze the data. **, *P* < 0.01.

It has been demonstrated that the Hippo signaling pathway can recruit macrophages, myeloid suppressor cells, and regulatory T cells by upregulating PD-L1 expression or stimulating cytokine production, inducing an immunosuppressive tumor microenvironment^27,28^. As an upstream inhibitor of the Hippo signaling pathway, CD44 can interact with the upstream regulatory factor Merlin of the Hippo signaling pathway, promoting the activation of the transcription enhancing cofactor YAP1 and subsequently activating the expression of downstream target genes^20,21^. To determine whether collagen I can promote downstream PD-L1 overexpression by activating the YAP1 signaling pathway during the development of keloid, the effects of different concentrations of collagen I on the protein expression of YAP1 and phosphorylated (p)-YAP1 in fibroblasts were evaluated. The results showed that, collagen I significantly promoted the protein expression of YAP1 in fibroblasts in a dose-dependent manner (Fig. 7A), while p-YAP1 was down-regulated by collagen I in a dose-dependent manner (Fig. 7B), indicating that collagen I activates the YAP1 signaling in fibroblasts. Moreover, under the premise of using YAP1 signaling pathway inhibitors, the effect of collagen I on downstream PD-L1 mRNA and protein expression was significantly reduced (Fig. 7C and D). These results suggest that collagen I promotes PD-L1 overexpression through activating YAP1 signaling in fibroblasts.

### CD44 is essential for collagen I in activating YAP1 signaling and promoting PD-L1 overexpression

**Fig. 8:**
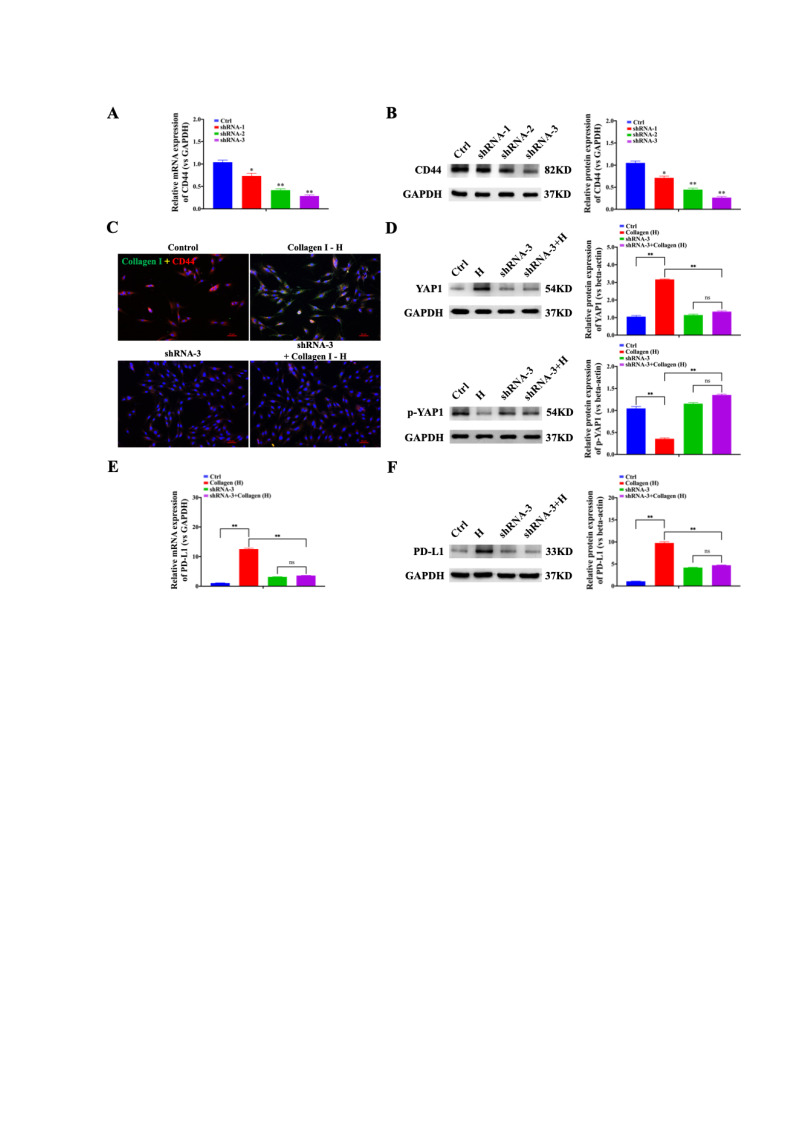
CD44 is essential for collagen I in activating YAP1 signaling and promoting PD-L1 overexpression. In this section, the primary fibroblasts were isolated from the human skin biopsies obtained from human keloid. (**A**) Real time PCR assay of CD44 mRNA in primary fibroblasts with CD44 silencing (shRNA-1, shRNA-2, shRNA-3). (**B**) Western blot assay of CD44 protein in primary fibroblasts with CD44 silencing (shRNA-1, shRNA-2, shRNA-3). (**C**) Immunofluorescence detection of collagen I and CD44 in primary fibroblasts that co-cultured with high concentration collagen I with or without CD44 silencing (shRNA-3). The cells were stained with antibodies against collagen I and CD44, respectively. Nuclear staining was done with DAPI. Scale bar, 50 μm. (**D**) Western blot and quantitative assay of YAP1 and p-YAP1 protein in primary fibroblasts that co-cultured with high concentration collagen I with or without CD44 silencing (shRNA-3). (**E**) Real time PCR assay of PD-L1 mRNA in primary fibroblasts that co-cultured with high concentration collagen I with or without CD44 silencing (shRNA-3). (**F**) Western blot and quantitative assay of PD-L1 protein in primary fibroblasts that co-cultured with high concentration collagen I with or without CD44 silencing (shRNA-3). Each experiment was performed three times independently. All results were shown as mean ± SD. Student’s t-test was used to analyze the data. **, *P* < 0.01.

Since CD44 is crucial for collagen I to promote PD-L1 overexpression, the present study subsequently focused on investigating the effects of collagen I on the YAP1 signaling pathway and downstream PD-L1 expression under CD44 silencing. The effect of different site interference plasmids on CD44 silencing was first observed, and it was found that all three interference plasmids could reduce the mRNA and protein expression of CD44, with interference plasmid 3 having the highest silencing efficiency (Fig. 8A and B). Subsequently, the co-localization of collagen I and CD44 under the premise of CD44 silencing was determined. High concentration of collagen I led to a high degree of co-localization of CD44 and collagen I, whereas, under the premise of CD44 silencing, the co-localization of these two molecules was significantly reduced (Fig. 8C). Next, the effect of a high concentration collagen I on the YAP1 signaling pathway under the premise of CD44 silencing was evaluated. The results showed that high concentrations of collagen I significantly promoted the protein expression of YAP1 in fibroblasts, however, CD44 silencing led to a significant decrease in the effect of high concentrations of collagen I on YAP1 expression (Fig. 8D). Meanwhile, high concentrations of collagen I significantly reduced the protein expression of p-YAP1 in fibroblasts, while CD44 silencing led to a significant decrease in the effect of high concentrations of collagen I on p-YAP1 expression (Fig. 8D). On the other hand, high concentrations of collagen I significantly promoted PD-L1 mRNA and protein expression, while CD44 silencing resulted in a significant decrease in the effects of high-concentration collagen I on PD-L1 mRNA and protein expression (Fig. 8E and F). Overall, the above results indicate that, CD44 is essential for collagen I-mediated activation of YAP1 signaling and promotion of PD-L1 overexpression in fibroblasts.

## Discussion

Keloid is a kind of skin fibrosis disease mainly characterized by the proliferation of fibroblasts. Its common pathological characteristics include abnormal proliferation of fibroblasts, excessive deposition of extracellular matrix protein and disorder of collagen fiber arrangement^12^. Certain studies have demonstrated significantly increased numbers of fibroblasts in keloid^5^, and markedly increased secretion of type I and III collagen by fibroblasts^12^. Various secreted neuropeptides combine with different skin cell receptors such as those located in keratinocytes, fibroblasts, mast cells and endothelial cells to further stimulate vasodilation, permeability, and release of histamine and cytokines (including TGF-β), which eventually leads to the continuous activation of fibroblasts and the formation of keloid^1^.

Notably, several signaling pathways were reported to be involved in the development and progression of keloid. Several studies have confirmed that the TGF-β/Smad signaling pathway plays an important regulatory role in the progression of fibrosis^29^. TGF-β1 could enhance α-SMA expression and increase the proliferation of fibroblasts in keloid tissues^30^. Song et al.^31^ reported that mechanical tension could lead to elevated expression of integrins αV and β3, causing increased collagen synthesis and fibroblasts proliferation. Gauthier et al.^32^ also demonstrated that wound tension could activate integrins, leading to increased collagen secretion and fibroblast proliferation. Gao et al.^33^ observed that the YAP/TAZ signaling pathway was abnormally activated in the fibroblasts of keloid tissues, which led to the enhanced expression of α-SMA and pro-fibrosis genes, as well as excessive deposition of matrix^34^. He et al.^35^ demonstrated that mechanical stretching enhanced the formation of hypertrophic scar via regulation of Piezo1-mediated calcium ion signaling.

The current study also confirmed that excessive deposition of collagen and abnormal proliferation of fibroblasts were the fundamental reasons for the continuous progression and recurrence of keloid. As the major constituent of extracellular matrix in the tissue microenvironment, collagen has been demonstrated to be associated with immune suppression and drug resistance in tumor-related research. In mice and human lung cancer, collagen deposition leads to a decrease in the total number of T cells and T cell failure, leading to resistance to PD-L1 blockers. Reducing collagen deposition can effectively increase T cell infiltration, reduce T cell failure and eliminate resistance to PD-L1^15^. In addition, previous studies have found that extracellular matrix deposition disorders, including collagen, are associated with inflammation, autoimmune diseases, and lymphoma. Immune cells with ITIM inhibitory receptors recognize collagen and negatively regulate immune responses^36^. Several studies have suggested that high concentrations of collagen can inhibit T cell activity and stimulate the immunosuppressive activity of macrophages^37–40^. To investigate whether a similar biological mechanism occur in the process of keloid formation, the present study revealed that there was a large quantity of collagen deposition in keloid tissue, and the total number of infiltrated CD8^+^ T cells was reduced, suggesting that the increase of collagen deposition may be closely related to the imbalance of the local immune microenvironment.

**Fig. 9:**
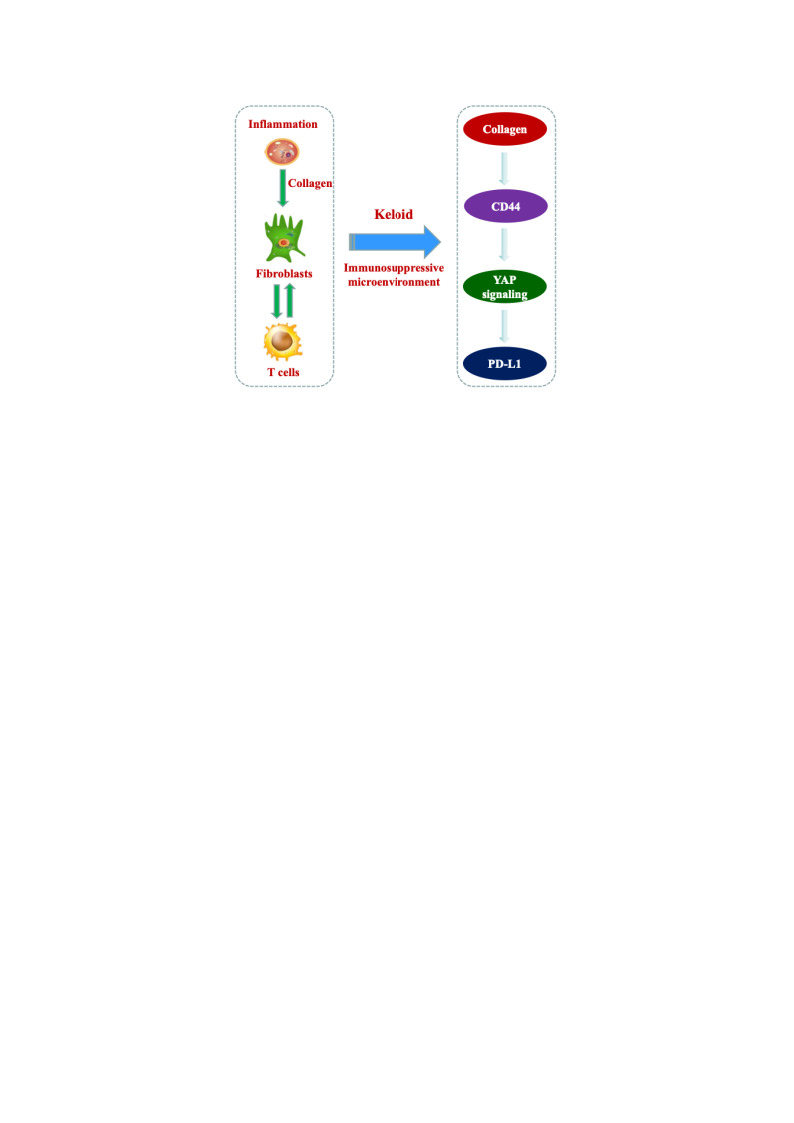
A schematic mechanism underlying the immumosuppressive microenvironment in keloid. In detail, inflammatory factors induced collagen deposition, which leads to the overexpression of PD-L1 and the formation of local immunosuppressive microenvironment by binding to the CD44 receptor of fibroblasts and activating the YAP1 signaling pathway, resulting in the inability to remove collagen deposition and the continuous proliferation of fibroblasts, and ultimately the formation of keloid.

Recent studies have found that there is persistent local inflammation in keloid, including a large number of increased inflammatory cells, fibroblasts, newly generated blood vessels and collagen deposition, accompanied by up-regulated expression of pro-inflammatory cytokines, and the mutual promotion between fibroblasts and local inflammation^1^. Inflammatory stimulation induced by trauma led to the continuous up-regulation of highly sensitive proinflammatory cytokines in the microenvironment, thus promoting the abnormal proliferation of fibroblasts and inducing keloid^41^. Overall, the aforementioned studies suggest that inflammatory reaction and immune system disorder may be the “originator” of keloid. From the perspective of pathobiology, keloid is similar to a chronic inflammatory disease with tumor tendency. Regardless of the type of inflammation induced by initial stimulation, from the perspective of the cell microenvironment of keloid formation, the local inflammatory response plays a key role in the regulation of fibroblast proliferation and collagen secretion in the process of scar formation^1^. In order to verify whether this phenomenon also exists in the keloid microenvironment, the expression of PD-L1, a costimulatory molecule that inhibits T cell activity, was detected in keloid tissue through pathological experiments in the present study. It was found that PD-L1 was significantly overexpressed in keloid fibroblasts, and was negatively correlated with the degree of infiltration of immune cells, including T cells, which suggests that local immunosuppression and immune escape occur in keloid tissue.

As a common type I transmembrane cell surface glycoprotein receptor, CD44 can be detected on the surface of lymphocytes and fibroblasts. CD44 signal activation is closely related to the abnormal proliferation of fibroblasts. For example, Osteopontin in renal tubular cell-derived exosomes can promote the abnormal proliferation of fibroblasts, as well as activate and aggravate renal fibrosis by binding CD44 in fibroblasts^18^. In the mechanism of scar formation in the elderly heart, CD44-positive MSCs could differentiate into fibroblasts, which was accompanied by collagen deposition and enhanced collagen fiber maturation^19^. It was found that CD44 could interact with extracellular viral matrix protein. It was reported that, in vitro, CD44 in the cell lysate could bind to the affinity column of type I and type VI collagen, and CD44 also allowed melanoma cells to effectively recognize types I and IV collagen^16,17^. Our study demonstrated that, collagen I could inhibit T cell proliferation by binding to fibroblasts and induce the formation of an immunosuppressive microenvironment. Moreover, the immunoinhibitory function of fibroblasts depends on their activation induced by interaction with collagen I; without stimulation from collagen at a sufficient effective concentration, fibroblasts maintain a resting state and do not exert obvious inhibitory effects.

The Hippo signaling pathway has been reported to recruit macrophages, myeloid suppressor cells and regulatory T cells by upregulating PD-L1 expression or stimulating cytokine production, inducing an immunosuppressive tumor microenvironment^27,28^. CD44 can interact with the upstream regulatory factor Merlin of the Hippo signaling pathway, promoting the activation of the transcription enhancing cofactor YAP1 and subsequently activating the expression of downstream target genes such as PD-L1^20,21^. The present study has shown that the collagen receptor CD44 is highly expressed in fibroblasts of keloid and co-localized with collagen deposition protein, which suggests that collagen deposition in keloid activates the signaling pathway mediated by the cell membrane receptor CD44. Further studies found that collagen could bind the CD44 receptor on the surface of fibroblasts, and promote the overexpression of PD-L1 through activating the YAP1 signaling pathway in fibroblasts. The results preliminarily revealed the molecular mechanism of PD-L1 overexpression in keloid tissue fibroblasts, and offered a promising therapeutic strategy with YAP1 signaling pathway and PD-L1 as a pivotal molecular target in keloid formation.

In summary, the present study preliminarily clarifies the biological mechanism of inflammation-induced collagen deposition, which leads to overexpression of PD-L1 and formation of a local immunosuppressive microenvironment by binding to the CD44 receptor of fibroblasts and activating the YAP1 signaling pathway, resulting in the inability to remove deposited collagen and the continuous proliferation of fibroblasts, which ultimately results in the formation of keloid (Fig. 9). Further in-depth exploration is expected to clarify the fundamental regulatory mechanism of local immune microenvironment imbalance for the continuous progression and recurrence of keloid, to provide a scientific basis for the complete clinical full understanding of the pathogenesis of keloid, and to aid in the future development of more effective drugs for the prevention and treatment of keloid.

## Supplementary Information

Below is the link to the electronic supplementary material.

Supplementary Material 1

Supplementary Material 2

Supplementary Material 3

## Data Availability

All data supporting the findings of this study are available within the paper and its Supplementary Information.
