# Supplementary material for: Collagen promotes PD-L1 overexpression in fibroblasts through binding to CD44 and activating YAP1 signaling during keloid formation
Source: Sci Rep. 2026 Jun 25;16:24025. doi: 10.1038/s41598-026-59337-6 (PMC13438748; doi:10.1038/s41598-026-59337-6)
Supplement: Supplementary file 2 [file 41598_2026_59337_MOESM3_ESM.docx]

**Figure S1.** A diagrammatic sketch of the signaling network in which the determined proteins are involved.
